# Vaccine Hesitancy in the Time of COVID-19: Attitudes and Intentions of Teens and Parents Regarding the COVID-19 Vaccine

**DOI:** 10.3390/vaccines10010004

**Published:** 2021-12-21

**Authors:** Amy B. Middleman, Judy Klein, Jane Quinn

**Affiliations:** 1 Department of Pediatrics, University of Oklahoma Health Sciences Center, Oklahoma City, OK 73104, USA; 2 Unity Consortium, Newtown, PA 18940, USA; judy@unity4teenvax.org (J.K.); jane@unity4teenvax.org (J.Q.)

**Keywords:** vaccine, vaccine hesitancy, COVID-19, social media, vaccine confidence, adolescents, teens, parents

## Abstract

To assess attitudes and intentions related to the COVID-19 vaccine during the pandemic, we surveyed adolescents aged 13–18 years and the parents of 13–18-year-olds using national research panels on three occasions or “waves”: before the COVID-19 vaccine was available, after it was available for adults, and after it was available for ages ≥12 years. Data on experiences with COVID-19, the importance of adolescent vaccines, and intentions regarding COVID-19 vaccination were analyzed across time points. We found that parental concerns about vaccine safety significantly increased from Wave 1 to 2. Social media had a negative influence on parents’ and adolescents’ opinions about vaccine safety. Demographic variables were associated with vaccination rates reported in Wave 3, consistent with known inequities related to vaccine access. Parents (70%) were supportive of concomitant COVID-19 vaccination with other adolescent vaccines for teens. It is important to address variables associated with vaccine hesitancy to increase COVID-19 vaccine coverage rates in the US.

## 1. Introduction

The first case of SARS-CoV-2 virus was reported in the United States in January 2020. Phase 1 clinical trials began in March 2020 for the mRNA-1273 (Moderna) vaccine and in April 2020 for the Pfizer/BioNTech mRNA vaccine [1,2]. As the race to develop an effective vaccine began, anticipation among scientists regarding the potential to create an effective and safe vaccine against SARS-CoV-2 was balanced by concerns that vaccine hesitancy might limit the number of people vaccinated when a vaccine became available. In 2019, the World Health Organization listed vaccine hesitancy as one of the top threats to global health [3]. Vaccine hesitancy is a complex concept, the etiology of which is context-specific across time, specific vaccines, environment, and culture [4]. It was difficult to predict the role vaccine hesitancy would play as the world grappled with controlling the global COVID-19 pandemic [5].

Since April 2020, many researchers across the globe have worked to assess vaccine hesitancy associated with a COVID-19 vaccine that was, at the time, not yet available. In the United States, a survey conducted in April 2020 among adults indicated that nearly 58% of those surveyed planned to receive a potential COVID-19 vaccine [6]. In a study from Finland, vaccine hesitancy seemed to increase as the time of the release of a vaccine drew closer; the proportion of adults aged 18 years and older agreeing to receive the vaccine went from 70% in April to 64% in December 2020, while those indicating that they somewhat disagreed, disagreed, or strongly disagreed with receiving the vaccine increased from 13% to 20% [7]. A small study among adults in Canada in August 2020 revealed that 20% and 12% of the population would not receive or were unsure if they would receive the COVID-19 vaccine, respectively [8]. In the United States in June 2020, a nationwide online survey in the U.S. revealed that 15% of adults reported they were unlikely to get vaccinated, while 23% were unsure [9].

Youths report a somewhat higher rate of acceptance of a potential COVID-19 vaccine. In China, 76% of youths surveyed from November 2020 to March 2021 indicated their acceptance of a future COVID-19 vaccine [10], and in the fall of 2020 80% of respondents from a public university in Canada said were willing to be vaccinated [11]. In the United States in October 2020, 76% of 14–24-year-olds surveyed expressed willingness to receive a COVID-19 vaccine (43% unconditionally willing and 33% conditionally willing) [12], and in March 2021 in the U.S. 76% of a sample of unvaccinated 18–25-year-olds indicated that they would receive a COVID-19 vaccine [13].

For adolescents under the age of 18 years, it is the attitudes of parents that most influences the likelihood of vaccination with the COVID-19 vaccine. Fewer studies have thus far explored the parental factors related to vaccinating adolescent children with a COVID-19 vaccine. A study in Italy revealed the overall acceptance of parents of children <18 years of age, with 60% reporting they would likely vaccinate their children [14]. In April 2020, 75% of adults in the U.S. reported willingness to vaccinate themselves, while 73% reported willingness to vaccinate their children [15]. Most recently, data from surveys presented at the Advisory Committee on Immunization Practices (ACIP) by the Centers for Disease Control and Prevention (CDC) in May 2021 indicated that 46–60% of parents plan to get their adolescent children vaccinated, with parents reporting a similar or somewhat lower intention to vaccinate their children versus vaccinating themselves [16]. In a survey conducted by the CDC and the University of Iowa on parents and adolescents in April 2021, 51% of adolescents of ages 13–17 years and 55% of parents of 12–17-year-olds indicated they would definitely or probably get vaccinated/get their adolescent vaccinated, respectively [16].

Vaccines are the most powerful tool available to help control the COVID-19 pandemic. Vaccine hesitancy could impair our ability to stem the spread of disease. In order to more fully understand vaccine hesitancy as it evolves across the time continuum of an ongoing pandemic, this study aimed to understand attitudes and intentions regarding preventive, routine vaccines and COVID-19 vaccines among adolescents and the parents of adolescents from 13 through to 18 years of age at three time periods or waves: in August/September 2020, before a COVID-19 vaccine was available for adults; in February 2020, after a vaccine was available for adults but before the vaccine was available to most teens; and in June 2021, after the vaccine was recommended for children 12–15 years of age.

## 2. Methods

A survey was developed among the members and liaisons of Unity, a consortium of individuals from academia; medical societies; government and public sector organizations; private sector industry; and numerous immunization, education, and youth advocacy groups working together to overcome barriers to adolescent and young adult vaccination. In addition to demographic data collection, the survey addressed three main areas of interest: (1) the participants’ experience with COVID-19 and the impact of the pandemic on their lives and activities, (2) attitudes regarding preventive health behaviors and routine immunizations, and (3) attitudes and intentions relating to COVID-19 vaccines. The survey was available in English. A third-party market research company, Dynata, was used to qualify participants from existing research panels who were either the parent or guardian of a 13–18-year-old or a parent or guardian willing to consent for their 13–18-year-old child to participate in an approximately 15 min online, self-administered survey. The parent and teen participants were not related as parent and child dyads. The teen respondents’ parents completed the initial demographic variables for teen participants; teen respondents completed the remainder of their survey. The research panels included a diverse sample population with respect to geography, race, ethnicity, age, education level, and household income. Participants were recruited via email. Dynata follows all national, regional, and local laws with respect to privacy and data protection. Dynata ensures panels comply with all applicable industry standards set by ESOMAR, MRS (UK), AMSRS (Australia), BVM (Germany), Insights Association (U.S.), and other international standards. The study was further approved by the Institutional Review Board at the University of Oklahoma Health Sciences Center.

Surveys were administered in three time “waves”: (1) before any COVID-19 vaccine was available, (2) after the COVID-19 vaccine was available to adults, and (3) after the COVID-19 vaccine was available to adolescents upon authorization for use in persons of age 12 and older. Wave 1 of the survey was conducted from 11 August through to 28 August 2020, for the teen survey; the parent survey data were collected from 11 August through to 18 September 2020, in order to achieve a greater participant diversity. Data for Waves 2 and 3 were collected between 4 February through to 1 March 2021, and 10 June through 30 June 2021, respectively. Survey items remained the same for all three waves, with the exception of additional items primarily related to whether parents (in Wave 2 and 3) and teens (Wave 3) had received the COVID-19 vaccine. The survey in Wave 3 also included a question pertaining to the concomitant administration of the COVID-19 vaccine with other recommended adolescent vaccines.

Surveys were hosted on a secure website and online participants were given a unique link to the questionnaire. Participant confidentiality was maintained with appropriate measures, including de-identifying participant responses from all stages of the study. Dynata attempted to re-survey participants from prior waves; Wave 2 included 39 repeat participants from Wave 1 and Wave 3 included 43 repeat participants from wave 2. Due to the low numbers of repeat participants, this variable was not included in the analyses.

For these analyses, items regarding experience with COVID-19 included whether the participant knew a person affected by the disease and how much the participants agreed or disagreed (5-point scale: strongly agree to strongly disagree) with several statements regarding their attitudes towards COVID-19. Items regarding routine vaccinations included rating how important specific vaccines are to your teen’s health (parent survey only; 4-point scale: extremely important to not at all important) and to what degree you agree or disagree with several statements regarding general attitudes about the safety and efficacy of vaccines (5-point scale: strongly agree to strongly disagree). Items regarding attitudes and intentions about potential COVID-19 vaccines included where participants go for information regarding COVID-19 vaccines, for what reasons participants would and would not get a COVID-19 vaccine for themselves/their teen (teen/parent survey), whether the parent participant would get themselves vaccinated and whether they would get their teen vaccinated (4 options: as soon as possible; under certain conditions, such as when it is convenient or after others have received it; if it were required for work/school; or do not plan to get the COVID-19 vaccine), and whether parents would have their teen receive other vaccinations at the same time as they received the COVID-19 vaccine. Demographic variables analyzed included: gender, age, age of teen child, urban/suburban/rural, household income, census bureau regions (Northeast, South, Midwest, West), race and ethnicity.

Data were analyzed by Blueberry Marketing and Sensory Research, a division of Reckner. For Wave 1 only, the participant sample was weighted to account for the lower participation of black respondents. Statistical analyses included frequencies and an analysis of variance (ANOVA). If the ANOVA showed significance, either a *t*-test or z-test was used for individual comparisons as appropriate for the data type. Significant differences were reported at the level of 0.05.

## 3. Results

For Waves 1, 2, and 3, there were 582/300 (weighted), 531/300, and 500/300 parents/teens responding, respectively. The participants are more fully described in Table 1. 

### 3.1. COVID-19 Experiences 

The proportion of participants who reported knowing someone who had been hospitalized for COVID-19, knowing someone who had died from COVID-19, and who had experienced themselves or a family member testing positive for COVID-19 increased significantly from Wave 1 to Wave 2 and stayed similar from Wave 2 to Wave 3. In Wave 1, 16%/15% of parents/teens knew someone who had been hospitalized with COVID-19, 13%/10% of parents/teens knew someone who had died from COVID-19, and 8%/6% had experienced themselves or a family member testing positive for COVID-19; in Wave 2, the proportions were 28%/25% of parents/teens, 23%/22% of parents/teens, and 20%/20% of parents/teens, respectively. Across all three waves, 82–86% of parents and teens strongly agreed or somewhat agreed that “COVID-19 is a serious disease,” while 57–63% felt “it is possible I will get infected with COVID-19”; there were no significant differences between parent and teen responses or responses across the waves (Table 2).

### 3.2. Routine Immunizations

**Figure 1:**
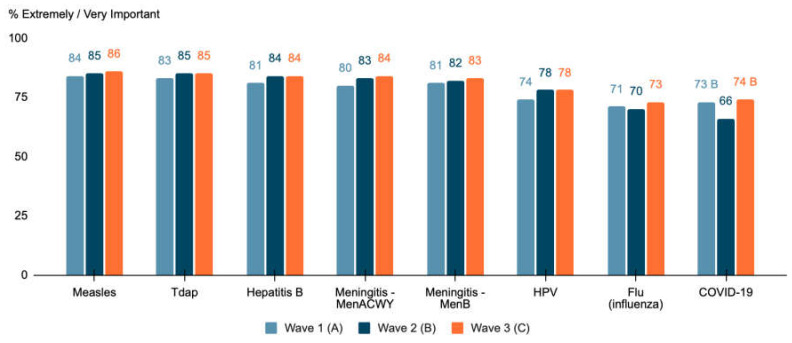
Parental rating of the importance of vaccines for teens. Question: How important is vaccination against these diseases to your teen’s health? Four-point scale, extremely important/very important/somewhat important/not at all important. Note: An upper-case letter indicates a significant difference (*p* < 0.05) in proportion from the column indicated by the letter.

Parent survey responses regarding the importance of routine vaccines and the COVID-19 vaccine (top box ratings of extremely or very important) are presented in Figure 1. COVID-19 vaccine and influenza vaccines were rated similarly by parents. Overall, parents reported a relatively high importance of getting the COVID-19 vaccine, although it was less than the importance reported for other routinely recommended vaccines. Over the three survey waves, 74%, 69%, and 78% of rural parents agreed that getting recommended vaccines for their teen was extremely or very important, significantly lower (*p* < 0.05) compared to the rates for suburban parents (82%) in Wave 1, urban (81%) and suburban (82%) parents in Wave 2, and urban (87%) parents in Wave 3. In general, a strong majority of both parents and teens supported the importance of vaccines for teen health (Table 2). The proportion of parent respondents reporting concerns regarding the safety and effectiveness of vaccines in general significantly increased over the course of the pandemic; overall, the majority of both parents and teens reported safety and efficacy concerns (Table 2). Of note, parents also reported agreement (which increased over the three waves of the study) that what they had read on social media about vaccine safety concerned them (with 41% reporting agreement in Wave 1 versus 51% in Wave 3), and, in Waves 1 and 2, teens reported greater concern with safety based on social media than parent participants did. Interestingly, over half of the teens surveyed reported feeling that if their peers were getting vaccinated, they, too, should get vaccinated (Table 2).

*COVID-19 Vaccines*: The majority of parents (70%, 71%, and 65% across Waves 1 through 3) reported their doctor or healthcare providers as the source they would use for COVID-19 vaccine information; this was the source of information that parents reported mattered the most, with public health or government sources being the second most common response. An increasing number of parents across the waves of the study reported seeking information from family and friends, schools, and social media (39%, 30%, and 31%, respectively, by Wave 3), although these sources were rated as being much less important. Across all three waves, parents and teens agreed on the reasons to get vaccinated against COVID-19, and the proportion of participants choosing each reason for getting the COVID-19 vaccine for themselves (teens) or their child (parents) remained approximately the same over time; one exception was that more parents in Wave 3 reported that having their teen feel safe around others was a reason to get vaccinated (Table 2). The most common reasons participants chose to get vaccinated (teens) or to have their child vaccinated (parents) included wanting to protect oneself and family members. The primary reason participants chose to *not* get vaccinated (teens) or not to have their teen vaccinated (parents) was concern about possible side effects, a concern that increased among parents and teens as the pandemic continued and vaccines were approved for use under the Emergency Use Authorization (Table 2). In general, teens reported feeling more concern regarding getting shots and injected with needles than parents did for their teens. Worries about having to pay for the vaccine decreased with time as it became increasingly clear that the vaccines in the United States were being administered at no cost. A greater proportion of parents in Wave 1 indicated that “the COVID-19 outbreak is not as serious as some say”; fewer parents chose that option in Waves 2 and 3 as the pandemic continued.

By Wave 3, 56% of the parents (278/500) had been vaccinated with at least one dose of COVID-19 vaccine themselves; parents identifying as Hispanic reported lower rates of vaccination (49%) compared to non-Hispanic parents (57%, *p* < 0.05), as did parents who reported being from both urban (46%) and rural (52%) areas versus suburban parents (67%, *p* < 0.05) and parents from the South (48%) versus parents from the Northeast (64%) and the West (60%, *p* < 0.05). Among parents, the percentage of those reporting not planning to get vaccinated themselves ranged from 14 to 24% (Table 2). Across all three waves of the study, a significantly higher proportion of parents (86 to 88%) who reported that the flu vaccine was extremely or very important for their teen’s health indicated that they would vaccinate their teen as soon as possible against COVID-19 when they were able (*p* < 0.05). In Wave 3, among parents who had not yet had their teen vaccinated, 33% reported not planning to get a COVID-19 vaccine for their teen; non-Hispanic (35% versus 26% for Hispanic parents, *p* < 0.05) and rural (46% for rural versus 26% for urban and 29% for suburban, *p* < 0.05) parents who had not yet vaccinated their teen were more likely to report that they did not plan to get their teen vaccinated. Race, household income, teen gender, and census region were not associated with parents not planning to get a vaccine for their teen. 

Fifty-eight percent (288/500) of parent respondents and 59% (176/300) of teen respondents reported that their teen or they, respectively, had received at least one dose of COVID-19 vaccine by Wave 3 (thus, 464/800, or 58%, of all teens referenced had been vaccinated). Factors associated with being vaccinated included: being from an urban community (67% of urban versus 56% of suburban versus 39% of rural teens were vaccinated, *p* < 0.05), Asian race (74% of Asian versus 54% of Black teens and 58% of White teens were vaccinated, *p* < 0.05), higher household income (38% from household incomes <$50,000/year versus 53% from household incomes $50,000–$99,000/year versus 73% from household incomes ≥$100,000/year were vaccinated, *p* < 0.05), living in the Northeast (77% of those from the Northeast versus 51% from the South, 55% from the Midwest, and 57% from the West were vaccinated, *p* < 0.05). Hispanic ethnicity and the age of the teen (median 15 years) were not associated with being vaccinated. 

**Figure 2:**
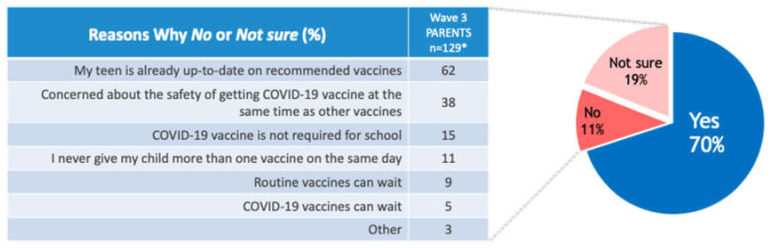
Parents’ willingness to have their teen get the COVID-19 and routine vaccines together. Survey question: The CDC and the American Academy of Pediatrics support giving other recommended childhood and adolescent immunizations at the same time as COVID-19 vaccines, particularly for children and teens who are behind on their immunizations. Are you willing to have your teen get COVID-19 vaccine and routine vaccines they may need at the same time? Survey follow-up question: Since you selected No or Not Sure, please select any of the following that explain your answer or add your own comment. Select all that apply; * reduced base = No/Not sure.

Seventy percent of parents were willing to vaccinate their teen with other vaccines at the same time as the COVID-19 vaccine (Figure 2); the most common reasons for not doing so were that the teen was already up to date on vaccinations (62%) and concerns regarding safety with concomitant vaccination (38%).

## 4. Discussion

This is the only study of which we are aware that surveyed teens and the parents of teens at three distinct times across 10 months of the COVID-19 pandemic regarding their attitudes, intentions, and behaviors related to COVID-19 and the COVID-19 vaccine. The study has demonstrated that despite knowing an increasing number of people affected by COVID-19, most people’s attitudes and intentions related to the COVID-19 vaccine did not change with time and were remarkably similar between the parents of teens and non-filial teens. Seventy six percent of parents indicated in August 2020 that they would get the vaccine and 78% indicated that would give the vaccine to their teen either as soon as possible or under certain circumstances, such as if they had time or made it a priority; this is consistent with intentions seen in other studies carried out among adults and adolescents [10,11,12,13,15]. The proportion of parents who indicated “I do NOT plan to get a COVID-19 vaccine” for themselves or their teens did not change significantly over the survey waves. While the proportion of parents and teens who had been vaccinated by Wave 3 was lower than those who reported an intention to get vaccinated, some of the unvaccinated were likely waiting for greater time availability or for others to be vaccinated first.

The significant changes noted over the 10 month span of the study were parents’ increased concerns in general about the safety and effectiveness of vaccines and that what parents had read on social media increased their concern regarding the safety of some vaccines. Along with these findings were increasing concerns about possible side effects of the COVID-19 vaccine, as the recommendations for the vaccination of first adults [17,18] and then teens (12 through 15 years of age) were announced [19]. As the pandemic continued, more parents and teens reported that “teens don’t get seriously ill from COVID-19.” It is important to note that the last wave of this survey was conducted before the Delta variant of the virus began to circulate widely in the United States; not as many children and adolescents were getting as sick from COVID-19 prior to the spread of the Delta variant of SARS-CoV-2 [20].

Concerns about vaccine safety have long been among the most common reasons for vaccine hesitancy. Multiple studies support the findings in this study in that those who are hesitant to receive a COVID-19 vaccine or give one to their children report safety as a top concern [12,13,16,21]. Some of these concerns regarding the COVID-19 vaccine include worry about how new the vaccines are and how rapidly they were developed [11,16,21,22]. Increased vaccine efficacy has also been found to be associated with increased intention to be vaccinated against COVID-19; in a study conducted in August and replicated in December of 2020 among 1000 participants in each iteration, the expected benefit of increased efficacy was found to be a strong positive determinant for intention to get vaccinated with a COVID-19 vaccine [12,23]. In addition, the author found no interaction effects between efficacy and safety concerns [23]. Both safety and efficacy concerns serve as major, independent determinants of vaccine hesitancy.

The role of social media, its perpetuation of misinformation, and the association with vaccine hesitancy have been reviewed in multiple papers [24,25,26]. Ruiz and Bell found that relying on social media was significantly associated with a lower intention to get vaccinated against COVID-19 [9]; the same finding was noted in a study of vaccine hesitancy among Italian parents conducted in December 2020 through January 2021 [14]. Loomba et al. found that exposure to misinformation on social media decreased the intention to vaccinate among participants from both the United States and the United Kingdom by over six percentage points [27]. The increasing proportion of parents noting in this study that what they had read on social media made them more concerned about vaccine safety is a worrisome finding.

In Wave 3, among parents who had not yet gotten their teen vaccinated, more rural parents and more non-Hispanic parents indicated that they do not plan to get the COVID-19 vaccine. Other studies have found differences in COVID-19 vaccine intention associated with these demographic variables. A Finnish study found that the factors associated with COVID-19 vaccine hesitancy varied with younger adult age, a finding also seen in other studies from the U.S. and Italy, implying that hesitancy may be associated with complacency among those under 50 years of age regarding somewhat the lower rate of disease in younger adults and the safety of vaccines in general [7,14,15,28]. Other studies investigating COVID-19 vaccine intentions have noted significant differences in vaccine intention based on race/ethnicity and socioeconomic variables. In a study of 911 14–24-year-olds, race predicted vaccine unwillingness (Black participants OR = 3.31 > White participants > Asian participants OR = 0.46, *p* < 0.001) [12]. Black respondents reported less willingness and Asian and Hispanic respondents greater willingness to get the COVID-19 vaccine in a study of 2279 U.S. adults in April 2020 [15]. Thompson et al. examined the association between race and potential COVID-19 vaccine uptake and found that Black adult study participants reported the highest medical mistrust scores compared to other racial groups [29]. This is not a surprising finding given historical context; Black Americans have experienced medical exploitation (e.g., Tuskegee Syphilis Study) and continued inequities in healthcare [30]. Hispanic parents in this study were less likely to have been vaccinated and more likely to consider vaccination for their yet-to-be-vaccinated teens. While this may seem contradictory, it may instead speak to access and equity issues [30]. Various studies differ regarding whether Hispanic parents report greater or less intention to vaccinate their children against COVID-19 than parents of other races and ethnicities [15,16]. It is not clear whether these data are confounded by access issues, which other studies have found to be associated with lower intent to vaccinate, including being without insurance, community type (urban versus rural), or lower household income [15,21,28].

In the end, it is vaccination data that provide the ultimate understanding of factors associated with vaccine hesitancy or refusal. In this study, teens from rural areas, of Black/African American race, from lower income households, and from regions other than the Northeast were less likely to get vaccinated with the COVID-19 vaccine within 2 months of it being available. The issue of vaccine hesitancy and refusal is complex; however, it will be critical to address all of these historical access and inequity issues and arrest the spread of misinformation as we work to vaccinate our entire nation against COVID-19. 

Despite these challenges, in this study healthcare providers remain the most relied upon source of COVID-19 vaccine information, a finding found in other studies of COVID-19 vaccination intention [11,28]. In addition, there was a relationship between understanding the importance of influenza vaccination and intending to receive the COVID-19 vaccine, an association that has also been described previously [9,31]. Additionally, the overall parental acceptance of the recommendation for concomitant vaccine administration with the COVID-19 vaccine is reassuring [32]. Although some parents in this study expressed worry regarding potential safety concerns, the majority of parents were accepting of the recommendation. This will help providers update any vaccines missed due to the pandemic as patients receive their COVID-19 vaccines. 

Vaccine hesitancy is a multifaceted and complex issue. There is likely not one single best way to address vaccine hesitancy. COVID-19 vaccine hesitancy seems to relate to many of the same themes as vaccine hesitancy seen with other routinely recommended vaccines. Hesitancy may ebb and flow based on perceived urgency and risk perception within the context of the pandemic [28]. Efficacy and safety concerns as well as distrust and concern regarding lack of transparency in our current vaccine development systems are always present and have been clearly articulated in recent U.S. Food and Drug Administration public commentary [22]. As with other vaccines, however, this study supports that provider recommendation is still most important factor in the decision to get vaccinated. Providers need to be armed with clear, concise communication strategies to address the misinformation about the COVID-19 vaccine that is often driven by uncertainty about safety and efficacy; lack of trust in those endorsing vaccination; and demographic characteristics associated with vaccine hesitancy, including lower education level, unemployment, younger age, and certain ethnic and racial groups that all lead to inequities in access to evidence-based information and healthcare [33]. The CDC (www.cdc.gov/vaccines, last accessed 18 December 2021) has developed copious materials for providers, including COVID-19 vaccine toolkits and FAQs, and the Immunization Action Coalition (www.immunize.org, last accessed 18 December 2021) provides resources for providers and parents. Professional organizations have engaged in and created multiple learning venues enabling providers to understand better how to teach parents and patients about vaccines [34]. It is critical for providers to use communication tools that help improve vaccine uptake in more routine times, including the use of presumptive language in vaccine recommendation [35,36,37]. One recent study performed in the UK found that written information, including a safety and efficacy statement; information directly addressing the personal benefits of vaccination; and, with less effect, information regarding the collective community benefits of COVID-19 vaccination, significantly decreased COVID-19 vaccine hesitancy among those identified on the Oxford COVID-19 Vaccine Hesitancy Scale as “strongly hesitant” [38]. Of course, because over half of the teen respondents in this study agreed with the statement “If my friends get vaccinated, I think I would need to get vaccinated, too”, incorporating an element of peer influence related to vaccination might also be of benefit in addressing teen vaccine hesitancy. These data provide an exciting opportunity to create effective materials for future health campaigns related to the COVID-19 vaccine and other vaccines. 

There are several limitations to this study. The study was conducted using research panels consisting of people interested in research, potentially creating a selection bias. This is demonstrated by the vaccination rate of 58% reported for teens by June 2021 in this study; national CDC data indicate that as of 31 July 2021, 42.4% of adolescents aged 12–17 years have had ≥1 COVID-19 vaccine [39]. The parent/guardian participants were not related to the teen participants, eliminating the ability to note possible concordance within parent–child dyads. Wave 2 included a somewhat more discordant demographic distribution of parents than noted in Waves 1 and 3, and these data were not weighted; however, data from wave 2 were not prioritized because the vaccine uptake data and intention to vaccinate teens who had not yet been vaccinated required a focus on data from Wave 3. These data were collected prior to the spread of the SARS-CoV-2 Delta variant; with increased rates of disease associated with this variant, the urgency of becoming vaccinated may have further changed the responses to these questions. 

Despite these limitations, this is the only study we have found that analyzed vaccine hesitancy across a 10-month continuum of the COVID-19 pandemic that included the introduction of vaccines for adults and for teens. Concerns associated with vaccine hesitancy seemed to increase from Wave 1 to 2 of the study and remained at a higher level by Wave 3, despite the availability of more information related to the COVID-19 vaccine’s safety. Factors associated with vaccine hesitancy with other vaccines seemed to predominate for this vaccine, including factors associated with access and equity related to preventive healthcare in general. Clear, concise, targeted messaging is needed to address the misinformation and distrust underlying vaccine hesitancy related to the COVID-19 vaccine; the health of the entire community is at stake. 

## Figures and Tables

**Table 1:** Demographic profile of the survey participants.

	Parents/Guardians			Teens		
	Wave 1	Wave 2	Wave 3	Wave 1	Wave 2	Wave 3
TOTAL Weighted	593	-	-	300	-	-
TOTAL Unweighted	582	531	500	300	300	300
**US Region (U.S. Department of Commerce, Economics and Statistics Administration U.S. Census Bureau)**						
Northeast	109	77	91	79	53	60
	19%	15%	18%	26%	18%	20%
Midwest	125	124	103	53	60	65
	22%	23%	21%	18%	20%	22%
South	215	202	193	98	121	94
	37%	38%	39%	33%	40%	31%
West	133	128	113	71	66	81
	23%	24%	23%	24%	22%	27%
**Gender**						
Male	264	176	215	133	165	152
	45%	33%	43%	44%	55%	51%
Female	314	354	285	164	131	144
	54%	67%	57%	55%	44%	48%
Non-binary/Other	0	0	0	2	3	2
	-	-	-	1%	1%	1%
Prefer not to say	4	1	0	1	1	2
	1%	*	-	*	*	1%
**Age of Parent/Guardian Participants**						
Age 25–34	34	34	33	-	-	-
	6%	6%	7%	-	-	-
Age 35–44	251	282	223	-	-	-
	43%	53%	45%	-	-	-
Age 45–54	203	160	159	-	-	-
	35%	30%	32%	-	-	-
Age 55–64	88	42	72	-	-	-
	15%	8%	14%	-	-	-
Age 65 or older	6	13	13	-	-	-
	1%	2%	2%	-	-	-
Mean	45.8	44.1	45.5	-	-	-
**Age of Teens**	**Age of the Parent/Guardian Participant’s Teen Child**			**Age of Teen Participant**		
Age 13	114	71	102	74	50	76
	19%	13%	20%	25%	17%	25%
Age 14	114	113	98	36	64	52
	20%	21%	20%	12%	21%	17%
Age 15	102	112	92	38	61	60
	17%	21%	18%	13%	20%	20%
Age 16	113	97	80	59	55	45
	19%	18%	16%	20%	18%	15%
Age 17	110	113	100	59	62	55
	19%	21%	20%	20%	21%	18%
Age 18	30	25	28	33	8	12
	5%	5%	6%	11%	3%	4%
Mean	15.1	15.3	15.0	15.3	15.1	15.0
Median	15.0	15.0	15.0	16.0	15.0	15.0
Std Deviation	1.5	1.5	1.6	1.7	1.5	1.6
Std Error	0.1	0.1	0.1	0.1	0.1	0.1
**Race**						
Black or African American	77	67	70	39	45	45
	13%	13%	14%	13%	15%	15%
American Indian or Alaska Native	15	2	5	8	2	2
	3%	*	1%	3%	1%	1%
Asian	15	20	25	8	11	6
	3%	4%	5%	3%	4%	2%
White	427	396	359	216	225	232
	73%	75%	72%	72%	75%	77%
Native Hawaiian or other Pacific Islander	4	4	1	8	0	1
	1%	1%	*	3%	-	*
Mixed race	15	19	20	8	8	9
	3%	4%	3%	3%	3%	3%
Some other race	15	19	17	8	6	5
	3%	4%	3%	3%	2%	2%
Prefer not to answer	15	4	3	8	3	0
	3%	1%	1%	3%	1%	-
**Hispanic Ethnicity**						
Hispanic	33	90	90	31	54	55
	6%	17%	18%	10%	18%	18%
Non-Hispanic	536	434	407	268	243	245
	92%	82%	81%	90%	81%	82%
Prefer not to answer	13	7	3	1	3	0
	2%	1%	1%	*	1%	-
**Community Type**						
Urban	199	167	207	109	128	145
	34%	31%	41%	36%	43%	48%
Suburban	284	242	199	134	120	110
	49%	46%	40%	45%	40%	37%
Rural	99	122	94	57	52	45
	17%	23%	19%	19%	17%	15%
**Household Income**						
Less than $50,000	138	228	134	95	113	72
	24%	43%	27%	32%	38%	24%
$50,000 to $99,999	173	155	141	76	85	93
	30%	29%	28%	25%	28%	31%
$100,000 or more	247	134	211	107	93	128
	42%	25%	42%	36%	31%	43%
Prefer not to answer	25	14	14	22	9	7
	4%	3%	3%	7%	3%	2%

* Signifies < 1%.

**Table 2:** Percentage of respondents reporting agreement with statements regarding COVID-19 experience, routine vaccines, and COVID-19 vaccines.

Topic	Parents/Guardians			Teens		
	Wave 1	Wave 2	Wave 3	Wave 1	Wave 2	Wave 3
	Column A	Column B	Column C	Column D	Column E	Column F
	Percentage choosing strongly agree/somewhat agree					
**COVID-19 Experience**						
COVID-19 is a serious disease.	82	83	86	83	82	85
It’s possible I will get infected with COVID-19.	62	63	60	60	59	57
**Routine Vaccines**						
It is important for all teens to get the vaccines recommended for them.	80 ^D^	78	77	72	73	74
I have some concerns about the safety of vaccines.	52	61 ^A^	63 ^A^	56	64	62
I have some concerns about the effectiveness of some vaccines.	54	60 ^A^	67 ^AB^	55	64	63
What I have read on social media has concerned me about the safety of some vaccines.	41	49 ^A^	51^A^	50 ^A^	56 ^B^	57
If my friends get vaccinated, I think I would need to get vaccinated, too.	-	-	-	58	54	63 ^E^
	Percentage choosing the option from “select all that apply”					
**COVID-19 Vaccines**						
**Reasons to NOT get the vaccine:**						
I am concerned about possible side effects.	41	54 ^A^	62 ^AB^	40	47	58 ^DE^
I am concerned my teen/I could get COVID-19 from the vaccine.	20	22	22	23	20	15
I do not think the COVID-19 vaccine will work well.	21	20	18	17	20	22
My teen does not/I do not like getting shots/needles.	12 ^B^	8	13 ^B^	20 ^A^	20^B^	21
I think the COVID-19 outbreak is not as serious as some say.	16 ^B^	11	14	12	14	14
I’m worried I/my parent might have to pay for it.	11 ^BC^	7	4	18 ^AF^	15 ^BF^	6
I think teens do not get seriously ill from COVID-19.	10	10	17 ^AB^	11	16 ^B^	19
**Reasons TO get the vaccine:**						
I want to protect my teen/myself.	51	55	54	53	49	51
I want to protect everyone in my family.	51	50	47	47	52	44
Vaccination is the best way for my teen/me to avoid a potentially serious disease.	39	40	43	37	38	38
I want to help protect my community.	39	39	36	35	36	34
Life will not go back to normal until most people are vaccinated, including teens.	38	39	35	37	35	34
My teen/I would be safe around other people.	34	36	41 ^A^	38	39	37
A family member is at high risk for COVID-19.	34	36^C^	29	31	35	30
	Percentage choosing one of the following options					
When COVID-19 is approved and available, which statement most closely represents what you will do for you? *						
Get vaccinated as soon as possible	30	28	8	-	-	-
Get vaccinated when I have time/make it a priority/others have safely been vaccinated	46	29	19	-	-	-
Only if required for my job/school	8	4	3	-	-	-
I have received at least one dose of vaccine	-	14	56			
I do NOT plan to get a COVID-19 vaccine	16	24	14	-	-	-
When COVID-19 is approved and available, which statement most closely represents what you will do for your teen? *						
Get vaccinated as soon as possible	28	29	12 ^^^	-	-	-
Get vaccinated when I have time/make it a priority/others have safely been vaccinated	50	58	42 ^^^	-	-	-
Only if required for my teen’s school	8	10	13 ^^^	-	-	-
I do NOT plan to get a COVID-19 vaccine	14	19	33 ^^^	-	-	-

Note: An upper-case letter indicates a significant difference (*p* < 0.05) in proportion from the column indicated by the letter. ^ The denominator for wave 3 included only those whose teen had not already been vaccinated (N = 212). * Significant differences between columns not reported due to different circumstances with respect to vaccine availability and, for response for teens, different denominator for wave 3.

## Data Availability

Data available upon request to Unity Consortium (unity@unity4teenvax.org).
